# Adding of apatinib and camrelizumab to overcome *de novo* trastuzumab resistance of HER2-positive gastric cancer: A case report and literature review

**DOI:** 10.3389/fphar.2022.1067557

**Published:** 2023-01-09

**Authors:** Huifang Lv, Yunduan He, Caiyun Nie, Feng Du, Xiaobing Chen

**Affiliations:** The Affiliated Cancer Hospital of Zhengzhou University, Henan cancer hospital, Zhengzhou, China

**Keywords:** trastuzumab, resistance, camrelizumab, apatinib, gastric cancer, case report, literature review

## Abstract

**Background:** Studies confirmed that trastuzumab plus fluorouracil-based chemotherapy improves the survival to more than 1 year in human with human epidermal growth factor receptor-2 (HER2)-positive advanced gastric cancer. However, there are still a small proportion of patients who do not benefit from trastuzumab treatment.

**Case summary:** Here, we described a case report of *de novo* trastuzumab resistance in HER2-positive gastric cancer. Concomitant cyclin-E1 (CCNE1) and HER2 amplification are associated with *de novo* trastuzumab resistance. Genomic analysis demonstrated CCNE1 amplification and TP53 mutation in a HER2-positive gastric cancer patient. This patient achieved significant survival benefit and good safety when the patient received triple regimens consisting of trastuzumab, apatinib, and camrelizumab.

**Conclusion**: Trastuzumab plus camrelizumab plus apatinib has the potential efficacy in HER2-positive gastric cancer patients who were previously treated with trastuzumab plus chemotherapy. This may lead to a new solution to trastuzumab resistance.

## Introduction

In the past decade, treatment based on precision medicine or individualized treatment has changed the outlook of many types of cancers (Nelson et al., 2021). Gastric cancer (GC) patients have evolved from treatment based on its molecular characteristics or its tumor microenvironment. Human epidermal growth factor receptor-2 (HER2) is closely related to the prognosis of gastric cancer (Lv et al., 2020). Since the advent of trastuzumab, survival has been significantly prolonged in advanced HER2-positive GC, and the treatment of GC has entered the era of targeted therapy (Bang et al., 2010). Unfortunately, a small proportion of patients could not benefit from trastuzumab treatment (*de novo* resistance). Even if someone benefits from the treatment, drug resistance to trastuzumab often appears after 1 year (acquired resistance). Once trastuzumab treatment fails, chemotherapy, including paclitaxel, docetaxel, or irinotecan, with or without ramucirumab is suggested to be the second-line treatment regimen (Hironaka et al., 2013; Wilke et al., 2014). New drugs have been explored in the second-line or aforementioned treatment of gastric cancer. Unfortunately, most of the clinical studies showed negative results (Ohtsu et al., 2013; Dutton et al., 2014; Bang et al., 2017). Antibody–drug conjugate (ADC) drugs have been successfully used in HER2-positive breast cancer (Modi et al., 2020; Montemurro et al., 2020). In addition to trastuzumab emtansine (T-DM1) failure in gastric cancer, the trials that involved trastuzumab deruxtecan (DS-8201a) and disitamab vedotin (RC48) have achieved positive results in gastric cancer (Thuss-Patience et al., 2017; Shitara et al., 2020; Xu et al., 2021). DS-8201a has been approved for the second-line treatment of gastric cancer by the US Food and Drug Administration (FDA), and ADC-RC48 is also used for the third-line treatment by the China National Medical Products Administration (NMPA).

Immune checkpoint inhibitors (ICIs) have changed the outcome of advanced tumors (Ribas and Wolchok, 2018). However, only 15 percent can benefit from programmed cell death 1 (PD-1) inhibitors alone. Therefore, the current research mainly focuses on the exploration of different immunotherapy combination treatment modalities (Hegde et al., 2016). Nivolumab combined with chemotherapy significantly improved the progression-free survival (PFS) and overall survival (OS) in the first-line treatment of HER2-negative gastric cancer patients (Janjigian et al., 2021), while in HER2-positive gastric cancer, pembrolizumab plus trastuzumab and chemotherapy recently showed a superior efficacy (Janjigian et al., 2020). In addition, a recent study showed that ICIs and anti-angiogenic drugs have synergistic effects on anti-tumor treatment (Galluzzi et al., 2016). The combination of immunotherapy and anti-angiogenic drugs has also been explored in gastric cancer. Regorafenib combined with nivolumab was approved to show survival benefits in patients with advanced gastric cancer in the REGONIVO study (Fukuoka et al., 2020). Lenvatinib plus pembrolizumab showed active anti-tumor activity in the EPOC1706 study (Kawazoe et al., 2020). However, most patients cannot afford them due to their high prices in China.

Apatinib, an anti-angiogenic drug, has been approved for the third-line treatment of GC and granted marketing approval by the NMPA (Li et al., 2016). Camrelizumab, the PD-1 blockades, has been approved effective in the treatment of lymphoma, esophageal squamous cancer, non-small cell lung cancer, and liver cancer by the NMPA (Nie et al., 2019; Qin et al., 2020; Luo et al., 2021; Zhou et al., 2021). In addition, camrelizumab has been approved as an orphan drug in hepatocellular carcinoma by the US FDA. Camrelizumab combined with chemotherapy followed by camrelizumab plus apatinib demonstrated an encouraging anti-tumor activity as the first-line therapy for patients with advanced adenocarcinoma in a phase II trial (Peng et al., 2021). Here, we described a specific case which apatinib and camrelizumab combined with trastuzumab could overcome trastuzumab resistance and primary chemotherapy resistance. This made a preliminary exploration of the treatment of triplet regimens (trastuzumab, camrelizumab, and apatinib) to overcome trastuzumab resistance in HER2-positive gastric cancer patients.

## Case presentation

**FIGURE 1:**
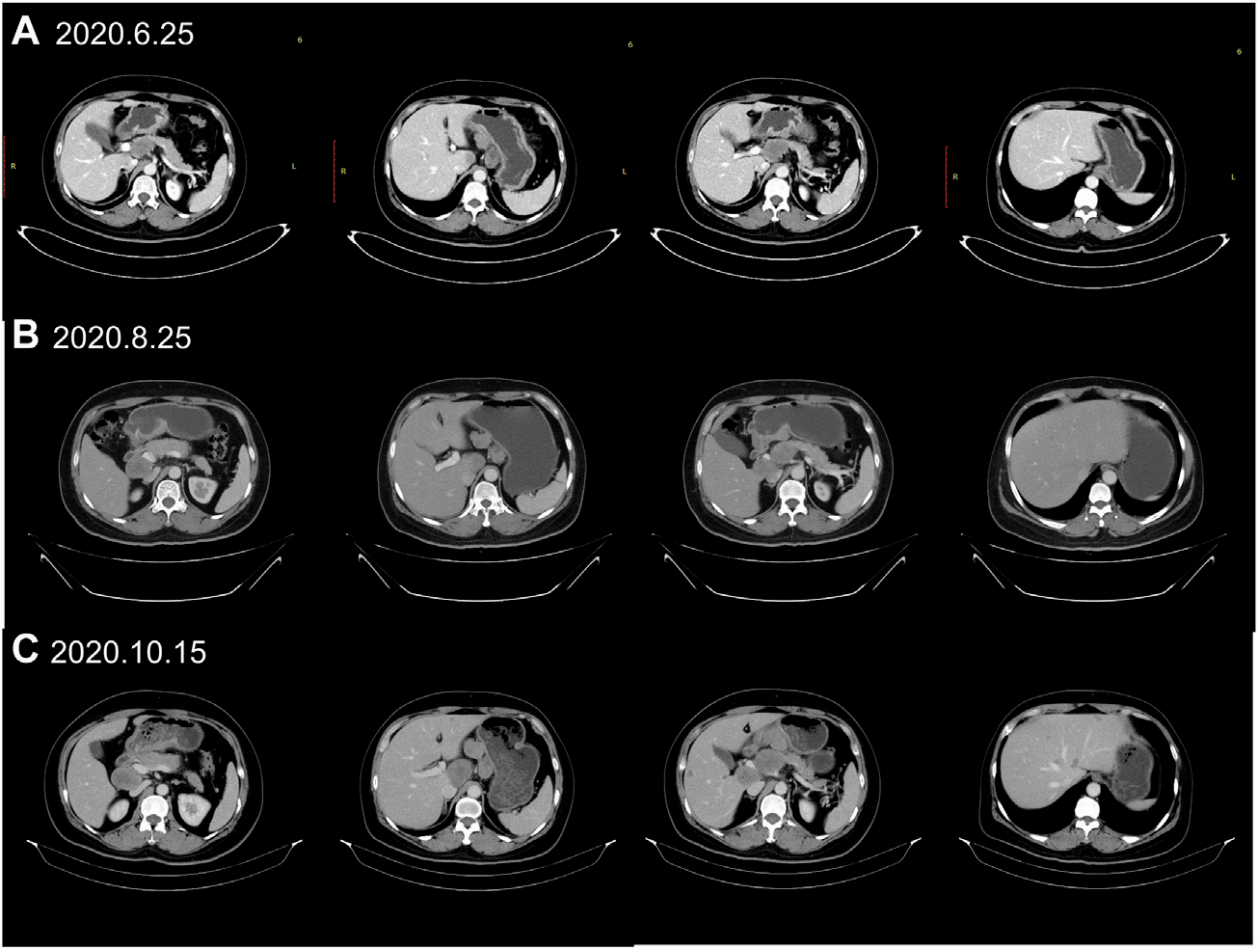
Treatment protocol before trastuzumab therapy. **(A)** CT scans (2020.6.25) showed the patient’s baseline disease; **(B)** CT scans (2020.8.25) demonstrated progressive disease (PD) in multiple lymph nodes metastases when first-line chemotherapy failed. **(C)** CT scans (2020.10.15) revealed new liver metastases when the second-line chemotherapy failed.

**FIGURE 2:**
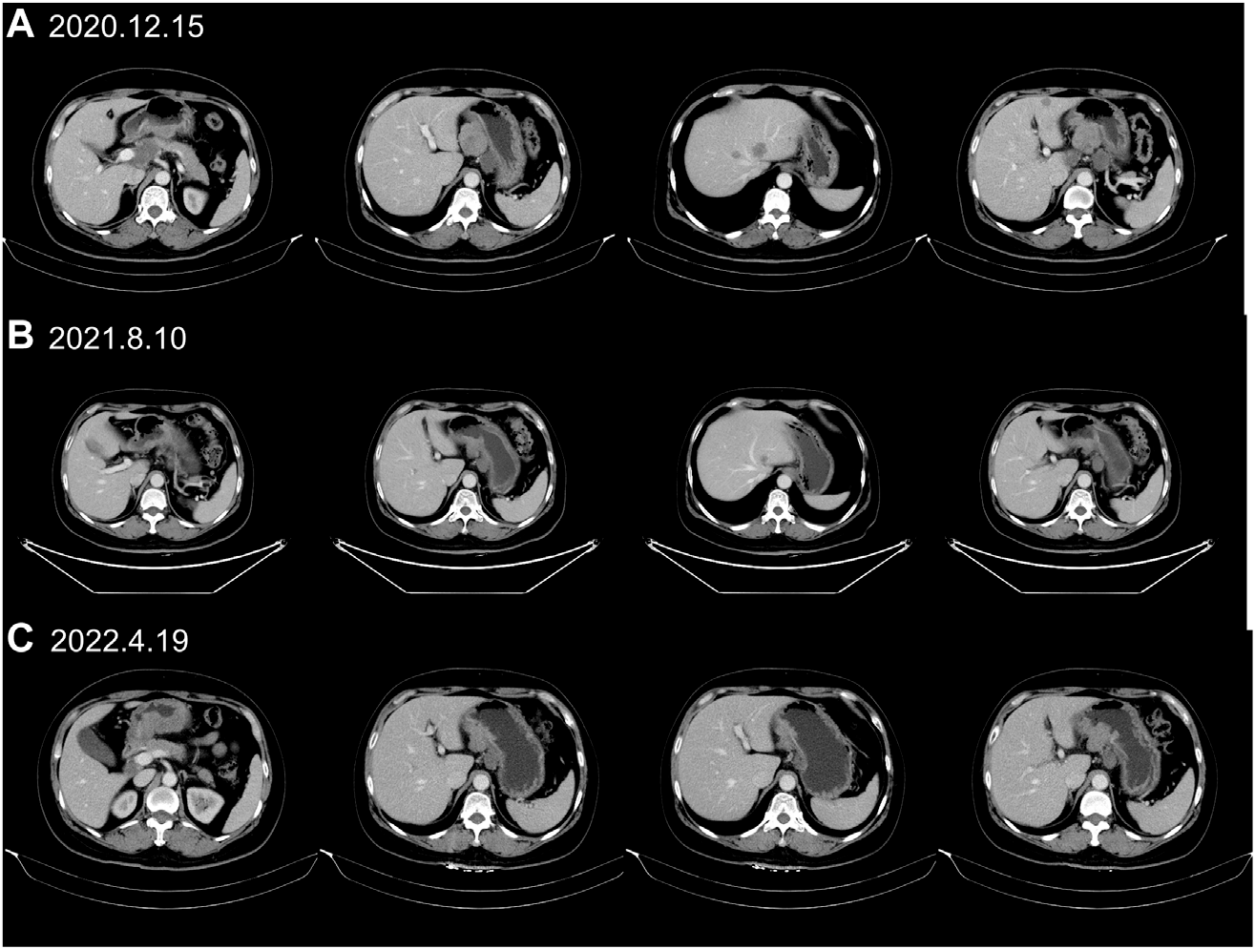
CT scans identified liver lesions during trastuzumab treatment. **(A)** CT scans (2020.12.15) demonstrated progressive disease (PD) in liver metastases after trastuzumab plus chemotherapy treatment. **(B)** CT (2021.8.10) showed that liver lesions had markedly shrunken 8 months after trastuzumab, apatinib and camrelizumab treatment. **(C)** The PR status (2022.4.19) continued with trastuzumab, apatinib and camrelizumab treatment until upper gastrointestinal bleeding happened.

**FIGURE 3:**
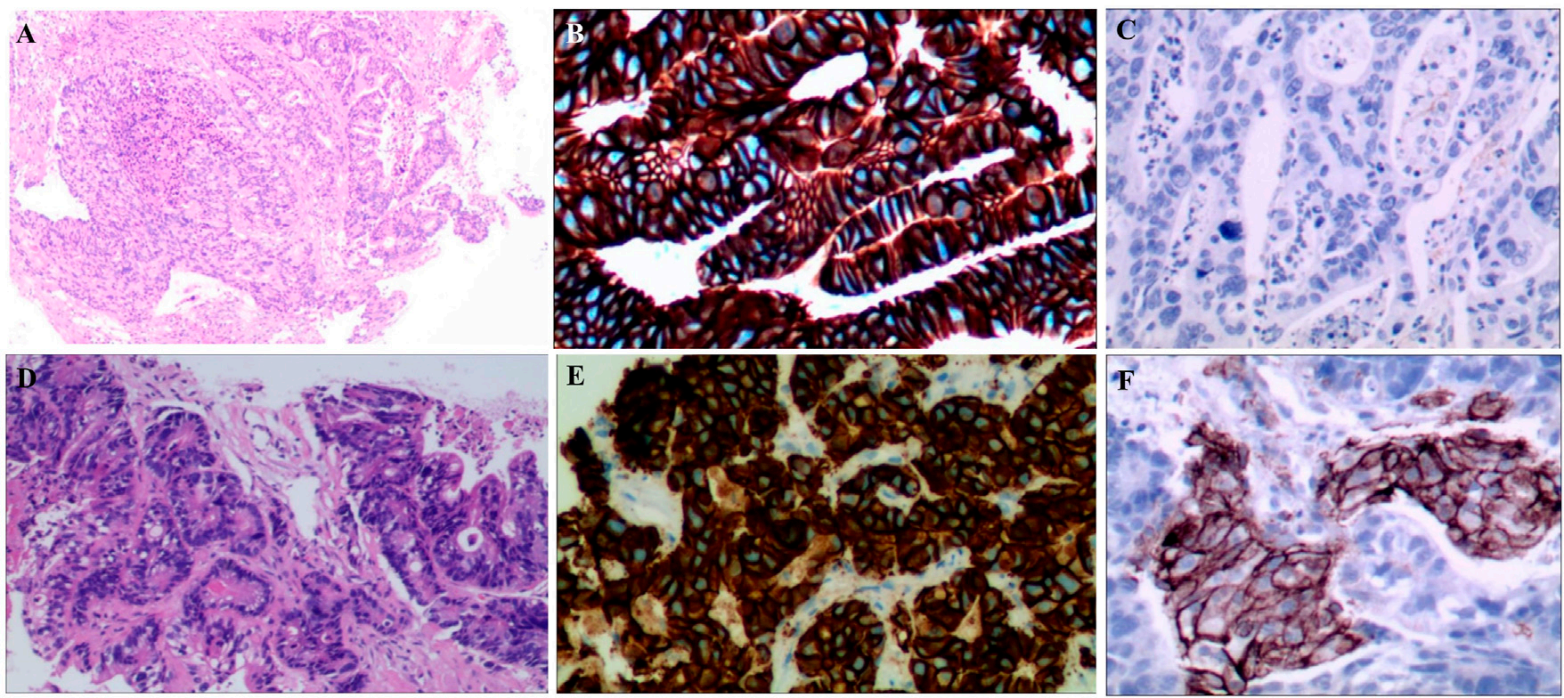
**(A)**. HE staining of gastric lesions (×400); **(B)**. HER2 positive on gastric cancer cells; **(C)**: PD-L1 negative on gastric cancer cells; **(D)**: HE staining of liver lesions (×400); **(E)**: HER2 positive on liver lesions; **(F)**: PD-L1 positive on liver lesions.

**FIGURE 4:**
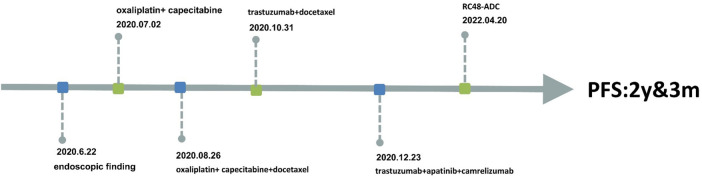
Information of this case report has been organized into a timeline.

A 55-year-old female suffered from abdominal pain. The information was as follows: KPS 90, H: 168 cm, W: 62 kg, and BSA: 1.7 kg/m^2^. She has no family history and genetic history. Gastroscopy showed a huge lump on the antrum of the stomach. The gastric biopsy revealed adenocarcinoma. CT (2020.6.25) scans demonstrated gastric antrum lesions with multiple metastases of hepatogastric and retroperitoneal lymph nodes (Figure 1A). The patient received two cycles of oxaliplatin plus capecitabine (oxaliplatin 200 mg d1; capecitabine 1.5 bid, d1–14, q21d). CT scans (2020.8.25) revealed disease progression in multiple lymph nodes (Figure 1B). Then, the patient was administered oxaliplatin plus capecitabine, along with docetaxel for one cycle (oxaliplatin 200 mg d1; capecitabine 1.5 bid, d1–14; and docetaxel 100 mg d1, q21d). New liver lesions appeared, and the primary tumor and lymph nodes increased (2020.10.15, Figure 1C). Then, the patient visited our hospital. Immunohistochemistry (IHC) revealed that tumor cells were HER2 3+ and negative for PD-L1 in gastric lesions (Figures 3A–C). The patient received trastuzumab plus docetaxel for two cycles. Trastuzumab was administered by intravenous infusion at a dose of 8 mg/kg on day 1 of the first cycle, followed by 6 mg/kg every 3 weeks. Docetaxel was administered at a dose of 120 mg on day 1 at 21 days intervals. CT scans (2020.12.15) showed increased liver lesions (Figure 2A). A multi-gene next-generation sequencing (NGS) testing was performed on liver lesions. Genomic profiling showed HER2 amplification (fold change, 49.2), cyclin-E1 (CCNE1) amplification (fold change, 16.0), and TP53 mutation (Table 1). IHC confirmed HER2 3+ and PD-L1 positive in liver lesions (Figures 3D–F). It is believed that CCNE1 amplification may be related to the resistance of HER2 monoclonal antibody and is associated with poor prognosis in HER2-positive gastric cancer. Then, the patient received trastuzumab, apatinib, and camrelizumab for eight cycles. Camrelizumab was intravenously administered with a dose of 200 mg at 21 days intervals. Apatinib was orally administered with a dose of 250 mg each day. The clinical efficacy was evaluated by imaging examination every 6 weeks. The optimal efficacy was partial response (PR) (2021.8.10, Figure 2B). During the treatment, the patient had moderate anemia and underwent two blood transfusions. The triple regimens continued until upper gastrointestinal bleeding took place, and the patient had to discontinue apatinib (2022.4.19, Figure 3C). Subsequently, she switched to ADC-RC48. A summary of her treatment history is illustrated in Figure 4. During the whole treatment, the patient provided written informed consent each time.

**TABLE 1:** Gene alterations in live metastasis.

Type	Result
NGS	
TMB	8.64 Muts/Mb
MSI	MSS
HER2 amplification	CN = 49.2
CCNE1 amplification	CN = 16.0
TP53 mutation	45.70%
IHC	
HER2	3+

## Discussion

Trastuzumab plus chemotherapy significantly improved the survival of gastric cancer (Bang et al., 2010), whereas most patients develop resistance about 1 year later. Overcoming trastuzumab resistance is still a difficult problem in the clinic until now. Different approaches have been explored recently in the second-line or aforementioned treatment of HER2-positive GC. Lapatinib, a small molecule tyrosine kinase inhibitor, blocks the downstream signaling of HER2. Adding lapatinib to paclitaxel cannot prolong PFS (3.7 m vs. 3.2 m, *p* = .33) and OS (10.2 m vs. 10.0 m, *p* = .20) in patients who progressed after trastuzumab plus chemotherapy in the TyTAN trial (Satoh et al., 2014). ADC drugs have dual antibody-dependent cell-mediated cytotoxicity (ADCC) and cytotoxicity effect. Due to bystander killing effect, DS-8201a had a better OS in patients who failed to respond to trastuzumab (12.5 m vs. 8.4 m, *p* = .0097) (Shitara et al., 2020). RC48-ADC had a similar result (OS = 7.6 m) (Xu et al., 2021). Margetuximab, an Fc-modified anti-HER2 agent, has more powerful ADCC. It demonstrated that margetuximab plus pembrolizumab has a synergistic anti-tumor activity, and better survival was achieved in patients who were previously treated with trastuzumab (OS = 12.9 months) (Catenacci et al., 2020). In addition, trastuzumab beyond progression was explored in HER2-positive gastric cancer. However, continuous use of trastuzumab plus switched chemotherapeutic agents after first-line treatment progression failed to improve PFS (3.2 and 3.7 months, *p* = .33) (Makiyama et al., 2020). However, an Ib/II trial illustrated that continuous use of trastuzumab plus ramucirumab and paclitaxel have a promising activity in patients who failed the first-line trastuzumab plus standard chemotherapy trial (OS: 13.6 months; PFS: 7.2 months) (Sun et al., 2021).

The mechanism of trastuzumab-acquired resistance has been explored in gastric cancer in recent years, but it is still unclear. It is considered that trastuzumab resistance is mainly related to HER2 receptor mutations, activation of MET, HER3, or FGFR receptors, or activation of downstream signaling such as PI3K/AKT and MAPK (Augustin et al., 2022). In contrast, substantially less effort has been devoted to investigating *de novo* trastuzumab resistance in gastric cancer. Gastric cancer was divided into four subtypes in The Cancer Genome Atlas (TCGA) project: tumors positive for Epstein–Barr virus (EBV); microsatellite unstable tumors (MSI); genomically stable tumors (GS), and tumors with chromosomal instability (CIN). A CIN subtype often harbors HER2 amplification, focal amplification of receptor tyrosine kinases (RTKs), and cell cycle regulatory gene amplification (Cancer Genome Atlas Research Network, 2014). It is believed that preexisting specific gene copy-number alterations that often co-occur with HER2 amplification might be co-drivers of tumorigenesis or may reflect intratumor heterogeneity, which would reveal the possible mechanism of *de novo* resistance to HER2-directed therapy (Kim et al., 2014).

*CCNE1*, one of the cell cycle regulatory genes, is overexpressed in multiple tumors. CCNE1 can lead to chromosomal instability which may contribute to tumorigenesis. In HER2-positive gastric cancer, patients with CCNE1 amplification have a poorer benefit than the patients without CCNE1 amplification. However, in the MSK cohort, there was no difference between patients with or without CCNE1 amplification (Janjigian et al., 2018). The result is controversial. Genomic analysis is often performed to identify the genetic alterations and is helpful for better selection of treatment regimens now. Previous studies showed that CCNE1 amplification is related to *de novo* trastuzumab resistance (Kim et al., 2014). In this case study, NGS testing demonstrated HER2 amplification, CCNE1 amplification, and TP53 mutation. Unfortunately, there is no specific drug for patients with CCNE1 amplification so far. In this case report, this patient achieved survival benefits when she received triple regimens consisting of trastuzumab, apatinib, and camrelizumab. She has a good quality of life and is very satisfied with her treatment regimen until now. This may lead to a new solution for trastuzumab resistance.

We have to admit that the mechanism of trastuzumab resistance is complicated, and the strategy in trastuzumab resistance is still an urgent problem to be solved in HER2-positive gastric cancer. The addition of apatinib and camrelizumab to trastuzumab has good efficacy and high safety in patients with *de novo* trastuzumab resistance. It needs to be further verified in clinical practices.

## Data Availability

The original contributions presented in the study are included in the article/Supplementary Material; further inquiries can be directed to the corresponding author.
